# Inconsistent Range Shifts within Species Highlight Idiosyncratic Responses to Climate Warming

**DOI:** 10.1371/journal.pone.0132103

**Published:** 2015-07-10

**Authors:** Daniel K. Gibson-Reinemer, Frank J. Rahel

**Affiliations:** Program in Ecology and Department of Zoology and Physiology, University of Wyoming, Laramie, Wyoming, United States of America; DOE Pacific Northwest National Laboratory, UNITED STATES

## Abstract

Climate in part determines species’ distributions, and species’ distributions are shifting in response to climate change. Strong correlations between the magnitude of temperature changes and the extent of range shifts point to warming temperatures as the single most influential factor causing shifts in species’ distributions species. However, other abiotic and biotic factors may alter or even reverse these patterns. The importance of temperature relative to these other factors can be evaluated by examining range shifts of the same species in different geographic areas. When the same species experience warming in different geographic areas, the extent to which they show range shifts that are similar in direction and magnitude is a measure of temperature’s importance. We analyzed published studies to identify species that have documented range shifts in separate areas. For 273 species of plants, birds, mammals, and marine invertebrates with range shifts measured in multiple geographic areas, 42-50% show inconsistency in the direction of their range shifts, despite experiencing similar warming trends. Inconsistency of within-species range shifts highlights how biotic interactions and local, non-thermal abiotic conditions may often supersede the direct physiological effects of temperature. Assemblages show consistent responses to climate change, but this predictability does not appear to extend to species considered individually.

## Introduction

Climate change has altered ecological phenomena across the globe [1],[2]. Meta-analyses of range shifts from climate change show a strong, generalized pattern of climate tracking with many species shifting their ranges uphill or poleward to stay within suitable climatic conditions [3]-[6]. Demographically, synchronous population fluctuations in response to climatic fluctuations (e.g., the Moran effect; [7]) have been documented as a consequence of rapid climate change [8],[9].

Temperature appears to be a dominant factor causing range shifts, although many factors influence species’ distributions [10]. A critical aspect of the most recent meta-analysis of terrestrial range shifts emphasized that warming was sufficient to predict the extent of range shifts: mean distances shifted and temperature increases were significantly related (r^2^ = 0.59 and r^2^ = 0.37 for latitudinal and elevational range shifts, respectively [4]). Thus, changes in species’ populations and ranges appear to be largely predictable on the basis of temperature when many species are considered together.

**Fig 1:**
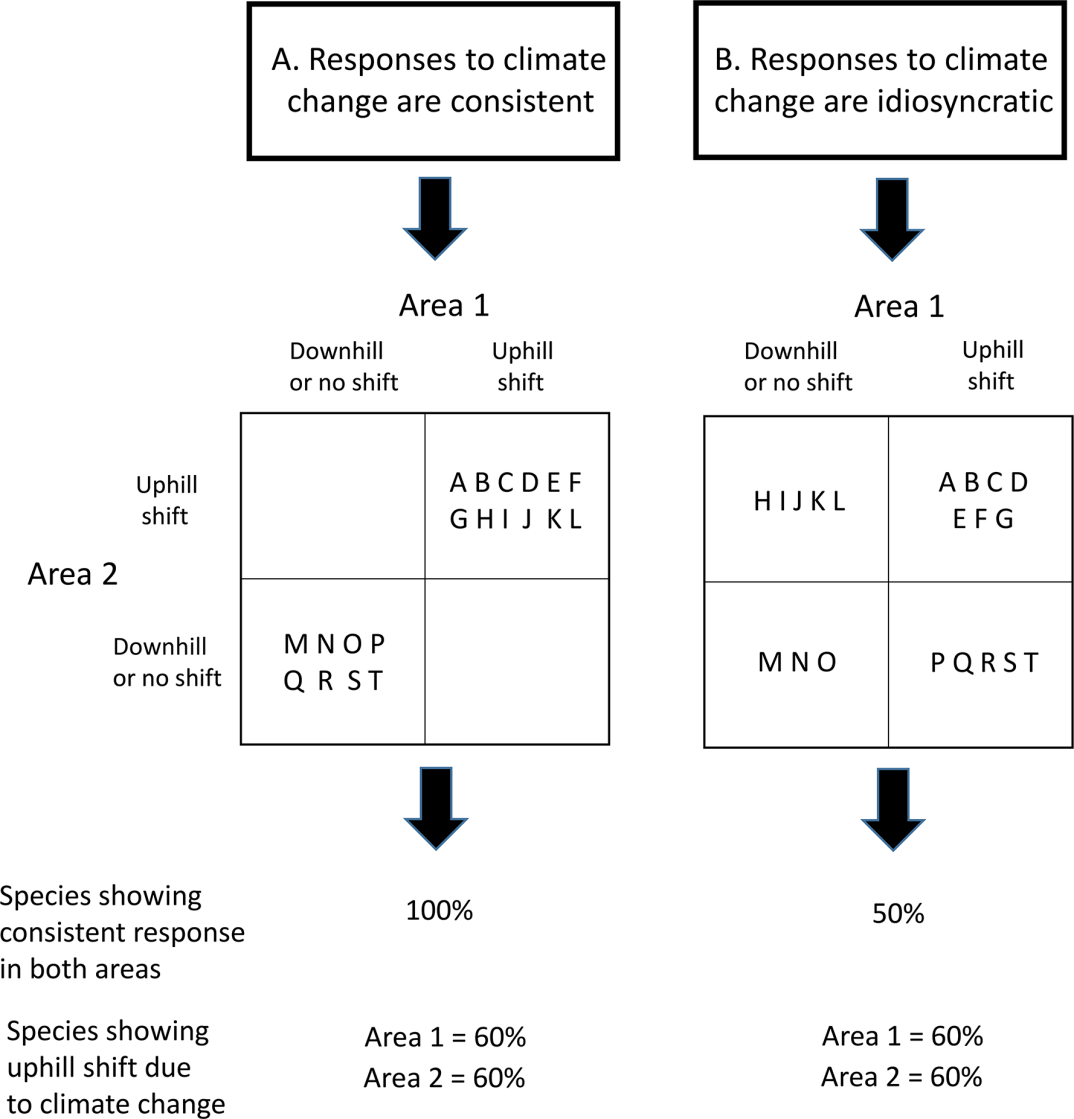
Different levels of within-species consistency can produce the same level of across-species consistency. (A) Consistent within-species range shifts from climate cahnge in two geographic areas versus idiosyncratic (B) within-species range shifts. For each scenario, the responses of 20 species (A through T) are depicted. In scenario A, 100% of the species show a consistent response between areas whereas in scenario B, only 50% of the species show a consistent response between areas. However, in both scenarios, 60% of the species show the expected uphill response to climate warming.

Prior analyses have focused on analyzing range shifts across species within the same area [4]. In essence, such approaches examine the consistency of range shifts while holding the environmental context constant and varying the species. A different approach is to examine the consistency of range shifts within species. If the species are held constant, but the environmental setting varies, how often will the same species show the same response? The key question in this analysis is whether species’ responses in one area are predictive of species’ responses in another area. Range shifts are caused by a variety of factors, but temperature, precipitation, species’ interactions, and dispersal are among the most important [11]. Temperature data are often available in conjunction with studies of range shifts, whereas data for precipitation, species’ interactions, and dispersal are less common. Here, we use paired populations of the same species, in different environmental contexts, where temperature is increasing in both situations. Few ecologists would suggest that temperature explains either all or none of the variation in species’ range shifts. However, how often warming temperatures lead to uphill or poleward range shifts within the same species has not yet been evaluated, and doing so would help ecologists understand the relative influence of temperature changes against a background of other ecological factors. If responses are consistent within species, then species moving uphill/poleward in one area should also be moving uphill/poleward in other areas. Similarly, species moving downhill/toward the equator in one area should also be moving downhill/toward the equator in different areas. As a result, few species will be moving in opposite directions between areas (Fig 1). Alternatively, factors such as species’ interactions or dispersal barriers may restrict species from tracking shifting climatic conditions.

An increasing number of studies document changes in species’ ranges as a consequence of climate change, providing the basis for meta-analyses that examine the consistency of responses to climate change across species [4],[5]. As the number of studies increases, some species have been measured in different geographic areas, providing the opportunity to examine responses to climate change within the same species. We identified species with documented responses to temperature increases in distinct geographic areas to evaluate the consistency of range shifts within species. We expected that the within-species consistency would meet or exceed the among-species consistency in range shifts associated with warming.

## Methods

### Selection of studies

We conducted a literature search to identify studies documenting climate-induced range shifts. We based our search on papers cited within two previous meta-analyses [3],[4] or any papers that have subsequently cited those studies, using Web of Science, in January 2014. Studies were excluded if they did not include information on individual species range shifts or if they did not include information on temperature trends within the study area; however, we included some studies that did not report temperature data in the original publication if temperature data for the area were included in a recent meta-analysis [4]. We also excluded species if their range shifts in different areas were assessed with different metrics (e.g., the upper elevational limit was measured in one area, and the optimal elevational limit was measured in another area). All paired comparisons therefore either changes at the upper limits or changes at a measure of the range centroid (e.g., mean or optimal elevation).

### Quantification of range shifts

The method of calculating range shifts in this analysis matches the methods used in a previous meta-analysis [4]. The range shift was measured as the difference between the recent range limit (or range centroid) and the historic range limit (or range centroid), with uphill or poleward shifts receiving positive values and equatorial or downhill shifts receiving negative values [4]. All studies included in the analysis experienced warming trends, eliminating the possibility that inconsistent range shifts could be caused by species shifting in response to different thermal trends. Both the magnitude of range shifts and the temperature change during the study period were based on the same values as a prior analysis, and the metric of distance shifted per years, standardized as m*year^-1^ for elevational shifts and km*year^-1^ for latitudinal shifts [4]. Thus, the values for range shifts and temperature change were standardized to reveal across-species predictability on the basis of temperature. We also standardized the range shifts by the rate of temperature change during the study period to account for different rates of warming in different areas. The resulting metric was distance shifted*°C^-1^, which incorporates the rate of range shift and the rate of temperature change:

$$\mathrm{distance shifted per}\;{}^{\circ}C=\frac{\left(\frac{\mathrm{distance shifted}}{\mathrm{year}}\right)}{\left(\frac{{}^{\circ}C}{\mathrm{year}}\right)}$$

### Consistency of the range shift

We evaluated the consistency of range shifts within species in two ways (full details on how we extracted data from each study are available in S1 File). Because the estimates of the rate of temperature change and the rate of range shifts are both subject to uncertainty, we first analyzed only the consistency of the direction in which species moved. The direction species shift is based only on the sign of the distance shifted (e.g., positive for uphill/poleward, negative for downhill/toward the equator), and this will be the same whether analyzed as distance*year^-1^ or distance*°C^-1^. There were three possible categories: species could move in the direction predicted by warming temperatures (uphill or poleward), opposite the direction predicted by warming (downhill or toward the equator), or show no change in their distribution (given as exactly 0 in published studies). Species with populations showing responses that fell into more than one of the above categories across geographic areas were classified as inconsistent. To provide a measure of inconsistency that used only shifts in opposite directions (e.g., uphill vs. downhill), we also analyzed a subset of the data in which all species with range shift of exactly 0 were removed.

We also examined the consistency of species’ range shifts (measured as distance*°C^-1^) by examining how well the magnitude of range shifts in one area predicted the magnitude of range shifts for the same species in another area. We did this by measuring the amount of variation in one area that could be explained by responses in another area, for which we used the coefficient of determination. It is possible that there are two classes of species: one that shows idiosyncratic responses to climate change and another that shows consistent responses to climate change. As another way of examining the predictive value of range shifts, we also analyzed the data when species that shifted in different directions were excluded. This method examined only species that shifted in the same direction to see if these species were also more consistent in the magnitude of their range shifts (measured as distance*°C^-1^) in different areas. We used the ratio of the larger range shift to the smaller range shift to estimate the difference in magnitude of range shifts, with a value of 1 representing identical range shifts in both areas.

Some species were measured in three different geographic areas. For these species, we created three paired comparisons to measure consistency. To compensate for the greater number of comparisons possible when a response was measured in three areas, we weighted the results for each study by the total number of species. For this reason, the total number of species examined is sometimes less than the number of paired comparisons.

**Fig 2:**
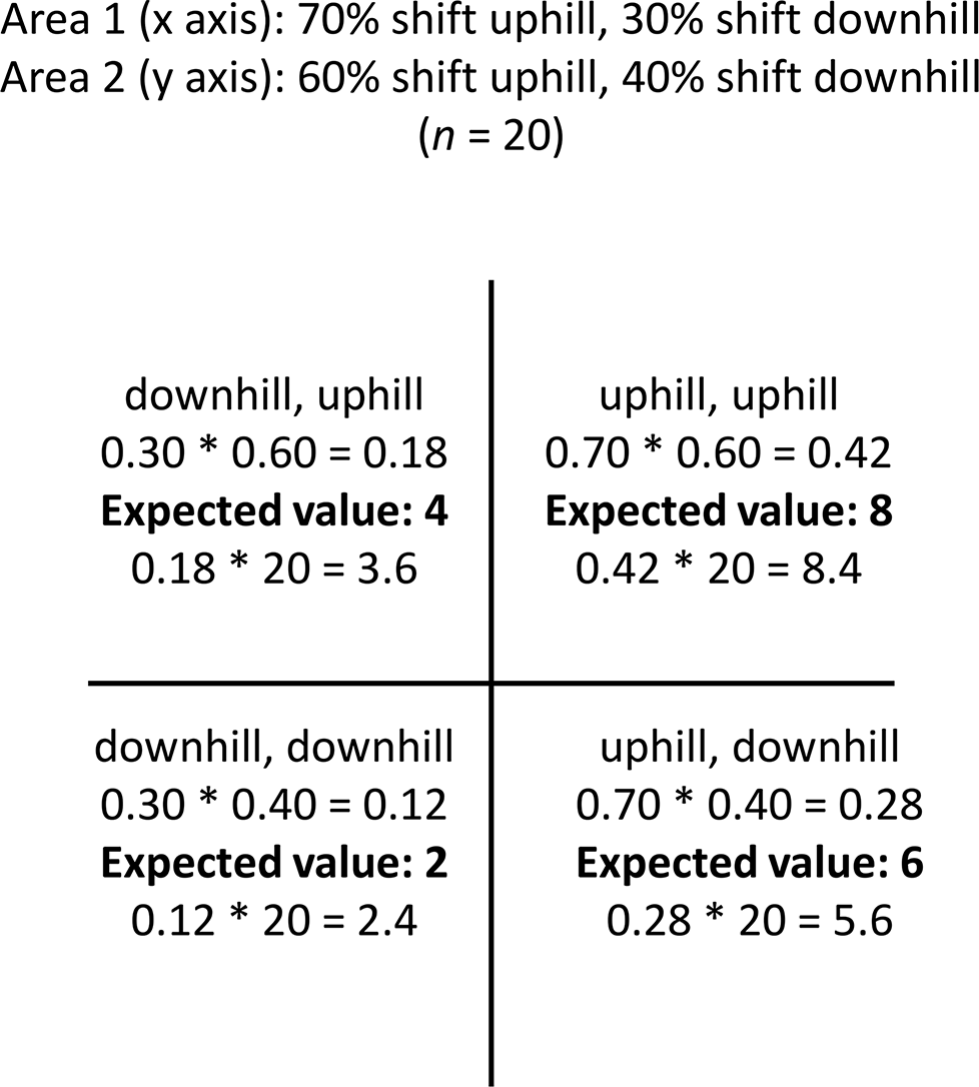
Derivation of expected values for the χ^2^ test. Each of four possible scenarios for paired range shifts are depicted.

We compared observed patterns of paired range shifts to expected values under a null model where species would shift independently in the two areas (i.e., idiosyncratically). There are four different ways a species can shift its range in paired areas: uphill in both areas, downhill in both areas, and two ways in which it can shift uphill in one area and downhill in another (Fig 1). The null model calculates the expected number of species shifting in these four ways based only on the overall proportion of species shifting uphill or downhill in each study (e.g., calculations for Areas 1 and 2 in Fig 2). Under this scenario, species identity has no influence on how species respond, and the direction any given species shifts is unrelated to how it has shifted elsewhere. Alternatively, if range shifts accompanying warming are consistent within species, there should be more species shifting uphill in both areas or downhill in both areas but fewer species shifting uphill in one area and downhill in another. We used a χ^2^ test in R (version 3.0.2 [12]) to determine if the observed number of species in each of the four quadrats in Fig 2 exceeded the null expectation (see Fig 2 for examples of how expected values were calculated). We omitted all paired comparisons in which there was a species range shift of exactly 0, as reported in the original study. We also omitted pairs of studies in which one study had all species range shifts in only one direction and the other study had species range shifts in both directions, as this produces a scenario in which the observed and expected values must be equal. We restricted the χ^2^ analysis to paired comparisons in which the expected values in each cell were five or higher in most cells, as this is a common practice for χ^2^ tests. This resulted in four paired comparisons. In two instances, the minimum expected values were four in one of the cells but we retained these analyses because the minimum values met or exceeded five in all other cells.

## Results

### Direction of Range Shifts

We identified 13 studies [11],[13–24] encompassing 273 species of plants, birds, mammals, and marine invertebrates across North America, Europe, and Australia with documented range shifts in two or three geographic areas (Table 1; data for all studies analyzed are presented in S1 File). Across all the species included in the analysis, 61% (426 out of 700 populations) shifted in the direction expected from warming, closely matching an estimate that 65% of species shift uphill with warming [25]. However, when we analyzed the species in paired comparisons, 50% shifted their distributions in categorically different directions in different geographic areas. The proportion of species shifting inconsistently was similar across plants, birds, marine invertebrates, and mammals (47%, 54%, 46%, and 60%, respectively). Omitting comparisons in which a species showed no range shift in at least one area, 42% of species moved in opposite directions (n = 257 paired comparisons). The observed number of species shifting in each of the four categories (uphill/uphill, downhill/downhill, uphill/downhill, or downhill/uphill) was not significantly different from the expected numbers under the null model in three of the four χ^2^ analyses (see S2 File).

**Table 1:** Studies used to compare the direction and magnitude of paired range shifts. The percentage of species with inconsistent range shifts is reported including all species, and the percentage of inconsistent range shifts when species with range shifts of zero are excluded is reported in italics; numbers of species in the analysis are reported in parentheses. Temperature trends are reported as rates, calculated by the total warming divided by the study duration or as the rate reported in the original study. Precipitation data, where available, were calculated in the same way. For each paired comparison, we list the coefficient of determination to describe the amount of variation in range shifts in one area that can be explained by range shifts in the other area. For both comparisons involving bird species, there are three paired comparisons summarized in each row; we report the mean value, weighted by the number of species. The overall weighted mean^**1**^ is listed as a footnote.

Taxa (region)	Paired studies	Percentage of species with inconsistent shifts (total number of species)	Temperature trends	Precipitation trends	Coefficient of determination (r^2^)	Minimum, maximum, and mean study duration	Type of range shift measured
Birds (France and Italy)	Archaux [13]; Popy [19]	50% (23); *50% (23)*	0.52°C/yr [13]; 0.091°C/yr [19]	None [13]; -5.2 mm/yr [19]	r^2^ = 0.02	11, 27, 20	Elevation: range centroid
Birds (California, USA)	Tingley [11]	47% (73); *36% (59)*	0.009°C/yr	0.1 mm/yr	r^2^ = 0.09	81, 98, 89	Elevation: upper limit
Mammals (California and Nevada, USA)	Moritz [16]; Rowe [20]	100% (5); *100% (1)*	0.030°C/yr [16]; 0.008°C/yr [20]	0.1 mm/yr [16]; 0.3 mm/yr [20]	r^2^ = 0.46	79, 88, 84	Elevation: range centroid
Marine invertebrates (Tasmania and Australia)	Pitt [18]; Poloczanska [21]	46% (11); *38% (8)*	0.022°C/yr [18]; 0.012°C/yr [21]	N/A [18]; [21]	r^2^ = 0.04	53, 58, 56	Latitude: poleward limit
Plants (Germany and Norway)	Bassler [23]; Felde [22]	71% (21); *71% (21)*	0.010°C/yr [23]; 0.010°C/yr [22]	1.5 mm/yr [23]; 5.7 mm/yr [22]	r^2^ < 0.01	81, 104, 93	Elevation: upper elevation
Plants (Germany and Switzerland)	Bassler [23]; Holzinger [14]	100% (2)*; 100% (2)*	0.010°C/yr [23]; 0.005°C/yr [14]	1.5 mm/yr [23]; N/A [14]	N/A (n = 2)	94, 104, 99	Elevation: upper elevation limits
Plants (Germany and Italy)	Bassler [23]; Parolo and Rossi [17]	71% (7); *50% (2)*	0.010°C/yr [23]; 0.031°C/yr [17]	1.5 mm/yr [23]; N/A [17]	r^2^ = 0.02	48, 104, 78	Elevation: upper elevation limits
Plants (France)	Bodin [23]; Lenoir [15]	47% (68); *46% (67)*	0.050°C/yr [23]; 0.035°C/yr [15]	No trend [23]; No trend [15]	r^2^ = 0.01	14, 22, 18	Elevation: optimum
Plants (Norway and Italy)	Felde [22]; Parolo and Rossi [17]	20% (5)*; 20% (5)*	0.010°C/yr [22]; 0.031°C/yr [17]	5.7 mm/yr [22]; N/A [17]	r^2^ = 0.22	48, 81, 65	Elevation: upper elevation limits
Plants (Switzerland and Italy)	Holzinger [14]; Parolo [17]	50% (46)*; 23% (30)*	0.005°C/yr [14]; 0.031°C/yr [17]	N/A [14]; N/A [17]	r^2^ = 0.02	48, 94, 70	Elevation: upper elevation limits
Plants (Switzerland and Norway)	Holzinger [14]; Felde [22]	33% (12); *27% (11)*	0.005°C/yr [14]; 0.010°C/yr [22]	N/A [14]; 5.7 mm/yr [22]	r^2^ = 0.01	81, 94, 88	Elevation: upper elevation limits

^1^ Weighted mean across all studies is 50% (42% when species with range shifts of exactly 0 are excluded).

We evaluated whether the proportion of species shifting inconsistently depended on how the range shifts were evaluated. As noted previously (Table 1), 50% of species displayed inconsistent responses when range shifts were based on changes in the cold-edge limits (upper elevation or poleward latitude). However, the results were nearly identical when range shifts were based on changes in the species’ range centroids (optimal or mean elevations), with 49% displaying inconsistent responses.

### Magnitude of range shifts

**Fig 3:**
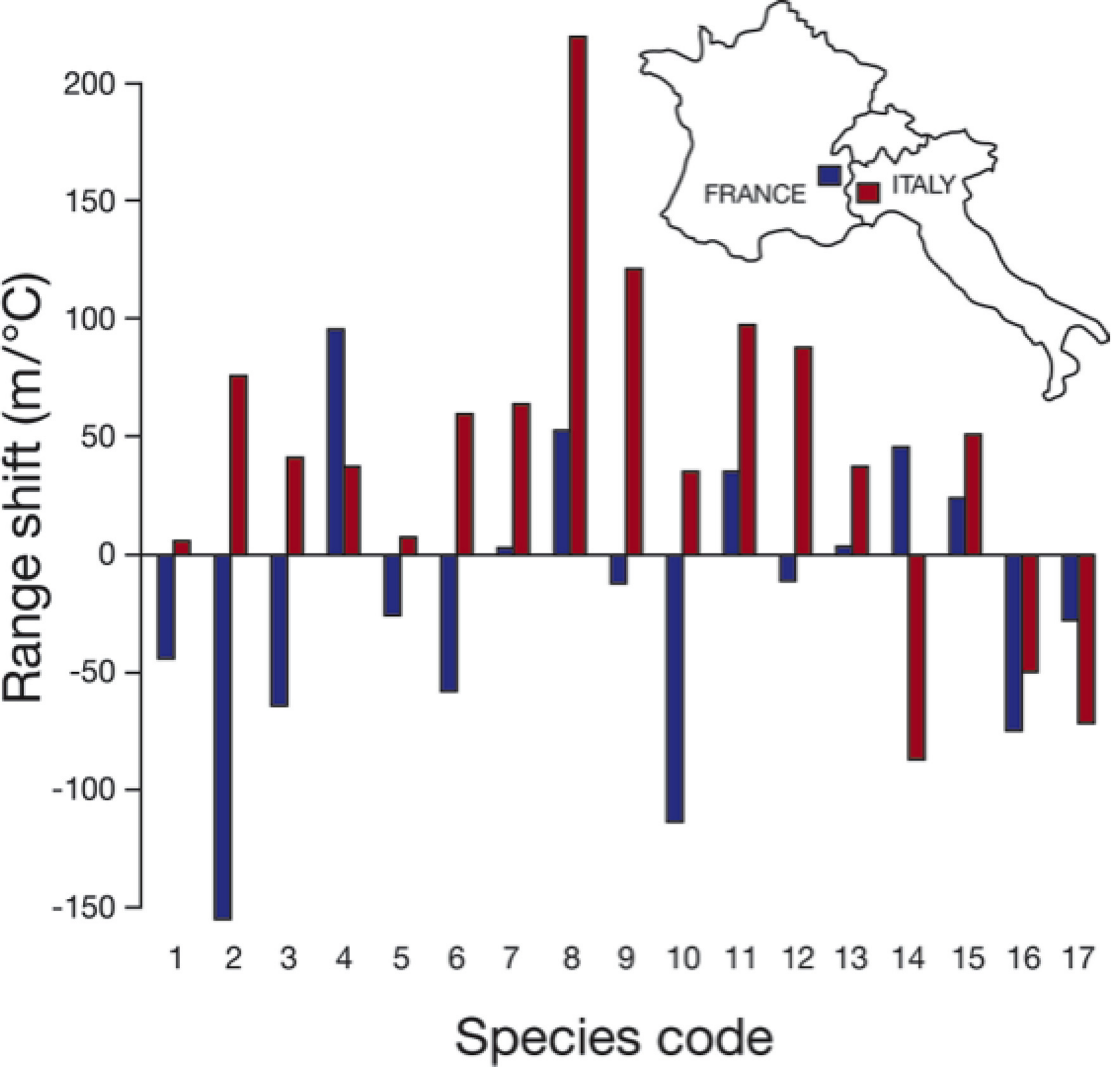
Idiosyncratic elevational range shifts among 17 bird species in France and Italy. Data from France (blue bars) are from [13], and data from Italy (red bars) are from [17]. Positive numbers indicate uphill shifts. Numbers on x axis refer to species codes given in the Supplementary Information (see S1 File for sources of data).

**Fig 4:**
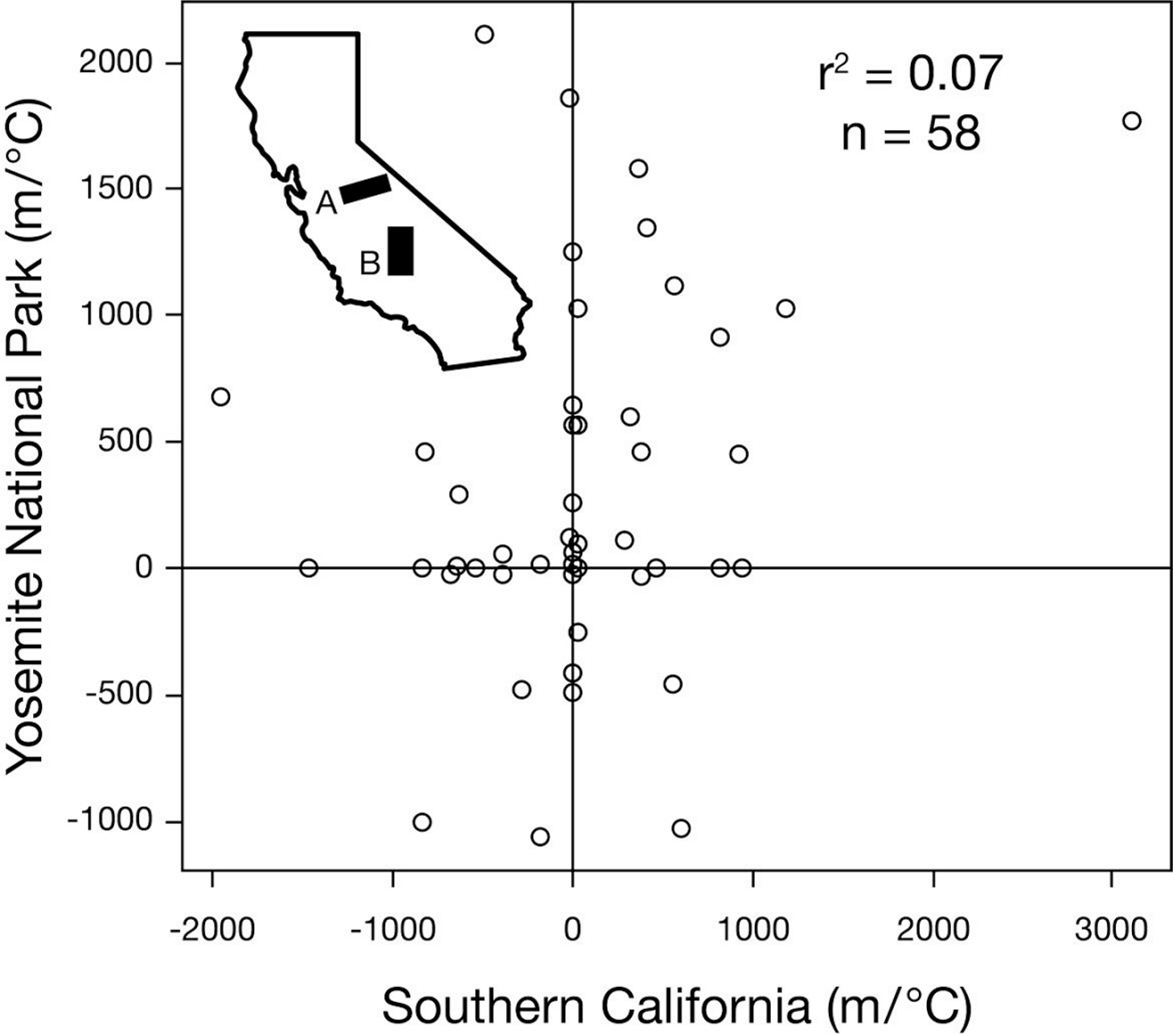
Elevational range shifts among 58 species of birds in California. Positive numbers indicate uphill shifts. Yosemite National Park is area A in the inset map, and Southern California is area B in the inset map. Both areas were reported in [11]. Scatterplots for all paired studies are available in the Supporting Information Figures (S1–S14 Figs).

When all 273 species were considered together, the weighted mean coefficient of determination was minimal (r^2^ = 0.05), as illustrated for birds in Europe (Fig 3) and birds in the southwestern U.S. (Fig 4; data for all comparisons are available in S1–S14 Figs). The results were not appreciably different when analyzed in terms of distance shifted per year, and we report the results as distance per degree of warming because we believe this better standardizes across areas with different levels of warming. Some species were consistent in the direction they shifted when measured in paired areas. For this subset of species, which included only species with non-zero range shifts, the mean ratio of the larger range shift divided by the smaller range shift was 8.8 (SD = 17.5).

## Discussion

The main result of this study is somewhat counterintuitive: climate-induced range shifts are generally predictable in their direction and magnitude, but this predictability declines substantially when the unit of analysis is an individual species, not a large group of species. This is a surprising result because a coherent pattern emerged from individual components that behaved idiosyncratically. The proportion of species showing inconsistent range shifts was fairly similar across a broad range of taxonomic groups (plants, birds, mammals, and marine invertebrates) and locations (North America, Europe, and Australia). Therefore, inconsistent range shifts seem to be a widespread response to climate change rather than a phenomenon in a single area or taxonomic group.

While previous research has highlighted the variability of species’ range shifts (e.g., [4],[25]), quantifying the within-species variability had not been accomplished at such a broad geographic scale. As such, the results do not have an empirical benchmark to which they are compared. On one hand, within-species consistency of 50–58% could be viewed as high. Range shifts are governed by many factors, and for a single factor to explain so much of how species shift is a testament to the importance of temperature. On the other hand, 50–58% within-species consistency also suggests that species’ responses to climate change will be modulated by other factors that do not necessarily co-vary with changes in temperature. We were unable to include any information from the world’s largest mountain ranges, in South American and Asia, because no data were available; therefore, studies that document paired range shifts in these areas would help to evaluate the cosmopolitan nature of the patterns we observed.

Consistent species-level responses (scenario A in Fig 1) would suggest information about species’ thermal physiology or other traits could sufficient for estimating species’ responses in areas without data on species’ range shifts. For instance, range shifts in an endangered species in one area could be used to estimate its response throughout its range, even where data on its distribution was scarce. We used χ^2^ analyses to test this idea. In three out of four χ^2^ analyses, the observed within-species consistency was more supportive of the null expectation that species respond independently between areas (scenario B in Fig 1). We also asked if variation in range shifts in one area can explain range shifts in another area by examining the coefficient of determination of range shifts in paired comparisons. Our results indicate that knowing a species’ range shift in one area provides little information about the magnitude of its range shift in a different area (e.g., Fig 4).

Some species were consistent in the direction they shifted when measured in paired areas. For this subset of species, which included only species with non-zero range shifts, the mean ratio of the larger range shift divided by the smaller range shift was 8.8. Even considering uncertainties in temperature measurements and range shift measurements, the observed ratio of 8.8 indicates there is almost an order of magnitude difference in the rate at which populations of a species respond to similar climate change. Further, this disparity exists for the subset of species that are moving in consistent directions, and up to half of all species are not even shifting in consistent directions.

### Ecological mechanisms to explain range shifts

Explaining the idiosyncratic nature of range shifts is an urgent question that we cannot fully assess using the data available; however, there are plausible explanations for the responses we observed. Two mechanisms, which are not mutually exclusive, may explain the inconsistency in range shifts. First, biotic interactions may exert a greater influence than climatic factors in determining distribution limits for some species. It is generally assumed that biotic interactions shape the warm-edge limits of species’ distributions and physiological responses to climate shape the cold-edge limits [26] (i.e., the poleward or uphill range limits). However, this may not result in uniform poleward or uphill shifts of species as temperatures increase. Theory suggests species with warm-edge limits (i.e., their equatorial or downhill range limits) set by competition may expand downhill when climate change acts a disturbance that decreases the strength of competitive interactions [25]. In addition, experimental manipulations have demonstrated that species interactions can override predictions of species’ responses to climate change. Species’ responses to experimental warming plots in a California grassland initially proceeded as expected based on physiological responses to temperature and water availability, but species interactions reversed the outcomes after several years [27]. Similarly, the effects of competition and predation in mesocosm experiments led to limits on species ranges that differed substantially from those expected based only on physiological responses [28].

As species track climate change to new regions, they may encounter species with which they had not previously interacted. In such novel assemblages, or groups of co-occurring species with no modern analog [29],[30], species’ distributions may be heavily influenced by strong biotic interactions. In the late Quaternary, North American mammals shifted in different directions during a period of climate change [31], similar to modern patterns documented here. In both cases, interactions between species whose ranges did not previously overlap may explain part of the inconsistency in species’ distributions. As more rapid climate change occurs in the 21st century, the occurrence of novel assemblages is likely to be greater, as well as the impact of novel interactions on species’ distributions.

A second mechanism that could explain inconsistent range shifts involves counter-gradients shifts such that species’ optimum climatic conditions move downhill during warming [11]. This type of response may be particularly likely to happen in ecosystems where water availability is more limiting than temperature. In such ecosystems, rising temperatures may be accompanied by increased precipitation, resulting in water availability isoclines moving downhill with warming. As a result, species are “pushed” uphill by rising temperatures but “pulled” downhill by greater water availability, as demonstrated in birds in California [11]. Precipitation may be the single most important factor in arid or Mediterranean ecosystems [11]. Therefore, species in arid ecosystems that display range shifts in opposite directions in different areas may be responding in a consistent fashion if the optimum climatic conditions are moving in different directions in different parts of their geographic distribution.

However, precipitation is unlikely to explain all of the variation observed in the present study. For instance, inconsistent range shifts occurred in marine ecosystems, where water availability is not an issue. Studies of marine [32] and freshwater [33] fishes have also shown counter-intuitive range shifts, suggesting range shifts opposite the direction expected by warming is a response that occurs even where water is not limiting. The lack of precipitation data in the studies analyzed here precludes any substantial analysis of this factor. Only in three instances were precipitation data available for direct comparison between studies; in all three instances, both temperature and precipitation increased in both areas, and these instances, the trend of species’ response was similar to the overall mean (Table 1). Examining the relative influence of temperature and precipitation is an important part of understanding range shifts, but our analysis was restricted to temperature.

Barriers to dispersal may account for some of the instance in which a population showed no range shift. Documenting barriers to disperal in studies of climate-induced range shifts is difficult, but a study of elevational range shifts in tropical moths identified geology as a potential barrier that may have prevented species from shifting their ranges uphill [34]. Moths use host plants, and their host plants may be unable to shift uphill if geological formations uphill of their current limits contain mineral formations that are unsuitable.

### Examining potential confounding factors

One potential explanation for species shifting in different directions between studies is that highly variable responses to climate change occur over short periods, but more consistent responses will emerge over longer timescales [1]. However, there was little correlation between the duration of studies, which ranged from a mean of 18 to 89 years between sampling intervals, and the proportion of species showing inconsistent range shifts (r^2^ < 0.01; S15 Fig). Thus, the temporal scale did not influence the results. A potential explanation for equatorial or downhill range shifts is that populations may be tracking local, counter-gradient temperature trends [6], so these directional shifts are actually consistent with the concept of climate tracking at local scales. However, the spatial scale in the analysis of within-species range shifts was the same as the spatial scale in the analysis of across-species range shifts. In both instances, the sources of data for range shifts and temperature change were the same, so the results cannot be explained by using different spatial scales for different analyses. It is possible that microclimatic refugia account for some of the species that did not shift their distributions in either direction (e.g., [35]), but further analysis of this was not possible with the data we analyzed.

In any analysis of published literature, there is concern that bias in the selection of studies can shape the trends observed. However, it is unlikely that selective publication or inclusion of studies is influencing the trends we measured. When range shifts are considered across species, the proportion of uphill or poleward range shifts closely matches previous estimates [4],[25]. Therefore, we are not analyzing a subset of species that show unusual responses but rather a large (*n* = 700 populations) group of species that are representative of climate-induced range shifts in general. Similarly, publication bias is unlikely to be influential in this analysis. We excluded studies that published single species accounts, as these are more likely to selectively focus on exceptional responses. Moreover, our analysis could only be done for species whose distributions had been studied in earlier time periods meaning the selection of which species are included was set by ecologists working several decades ago, before climate-induced range shifts were a consideration.

### Predictable assemblages, unpredictable populations

We interpret the collective results as evidence that temperature is sufficient to predict mean range shifts across an assemblage of species but insufficient to explain the results at the level of individual species. Each method of analyzing range shifts within species produced similar results. At the broadest level, the direction species moved was frequently inconsistent. The magnitude of a population’s range shift in one area was a poor predictor of the magnitude in another area, and even for the subset of species shifting in the same direction, there was about an order of magnitude difference in the response. The χ^2^ test indicates the populations appear to be much less predictable than assemblages. This implies that rising temperatures act as a force pushing most species to track temperatures, although which species do so in an area is strongly influenced by non-thermal factors.

Importantly, the results we observed do not contradict previous meta-analyses: most populations (61%) in our study shifted in the direction expected from warming, which agrees well with estimates from other analyses [4], [25]. An intriguing aspect of climate-induced range shifts is that a coherent pattern emerges when species are considered collectively even though species appear to behave less predictably when considered individually (e.g., scenario B in Fig 1). Although it is a fundamental premise that species’ physiological requirements will drive their response to climate change [36], the low within-species consistency in response to rising temperatures suggests biotic interactions and local climatic conditions may often overwhelm the influence of temperature, at least at the warming rates observed in the 20th century. While we expected that non-thermal conditions would be somewhat influential in shaping range shifts, we were surprised at how influential they appear to be for the studies we examined.

### Conservation implications

If species shift their distributions one way in one region and shift their distributions in another direction in a separate region, what is the effect on their rangewide distribution? Presently, there is little evidence to suggest changes in geographic distribution in a single area could be used to extrapolate to a species’ entire range. Studies documenting changes in distribution throughout a species’ entire range are rare (but see [37],[38]). Consequently, many of the inferences regarding climate change impacts on species’ distributions come from transect studies, partly because mountain transects contain large temperature gradients over a short distance and partly because these are often the best baseline data from past decades. Yet, the results of this analysis suggest that measuring changes in distributions in a single area may provide little information about the total change in a species’ distribution as a consequence of climate change. Much of the knowledge regarding climate-related range shifts comes from syntheses of many studies [4],[39], most of which do not document range shifts across a species’ entire range. The present research suggests that the most general conclusions of range shift syntheses are correct (most species in any given area shift to cooler regions), but what happens along a transect may not be representative of what happens at a much larger spatial scale, such as an entire mountain range.

This study highlights a problem for managing and conserving biodiversity at the level of an individual species, as is often mandated through laws such as the Endangered Species Act of the United States. Forecasting will probably work well for assemblages, because the signal from warming becomes apparent when averaged across a large numbers of species. But forecasting may be less accurate for single species since local, non-thermal climatic factors and species interactions may override the effects expected due to regional warming. Similarly, projections of temperature trends for the 21^st^ century may be less variable than projections of precipitation trends. Locally-refined estimates of climate change and incorporation of biotic interactions will produce better predictive frameworks but will require more effort and cost to collect the necessary data [40],[41].

## Supporting Information

S1 File Sources and method for calculating range shifts for all studies included in the analysis. (DOCX)

S2 File Data, code, and calculations for chi-square analysis. (PDF)

S1 Fig Scatterplot of elevational range shifts in birds at Mont Ventoux and the Giffre Valley, Switzerland [13]. (TIFF)

S2 Fig Scatterplot of elevational range shifts in birds at the Giffre Valley, Switzerland [13] and Alta Valsessera, Italy [19]. (TIFF)

S3 Fig Scatterplot of elevational range shifts in birds at Mont Ventoux, Switzerland [13] and Alta Valsessera, Italy [19]. (TIFF)

S4 Fig Scatterplot of elevational range shifts in birds at Lassen National Park, California, USA, and southern California, USA [11]. (TIFF)

S5 Fig Scatterplot of elevational range shifts in birds at Yosemite National Park, California, USA, and southern California, USA [11]. (TIFF)

S6 Fig Scatterplot of elevational range shifts in birds at Yosemite National Park, California, USA, and Lassen National Park, California, USA [11]. (TIFF)

S7 Fig Scatterplot of elevational range shifts in mammals in California, USA [16] and Nevada, USA [20]. (TIFF)

S8 Fig Scatterplot of latutidinal range shifts in marine invertebrates in Tasmania [18] and Australia [21]. (TIFF)

S9 Fig Scatterplot of elevational range shifts in plants in Germany [23] and Norway [22]. (TIFF)

S10 Fig Scatterplot of elevational range shifts in plants in Germany [23] and Italy [17]. (TIFF)

S11 Fig Scatterplot of elevational range shifts in plants in southern France [23] and western France [15]. (TIFF)

S12 Fig Scatterplot of elevational range shifts in plants in Norway [22] and Italy [17]. (TIFF)

S13 Fig Scatterplot of elevational range shifts in plants in Switzerland [14] and Italy [17]. (TIFF)

S14 Fig Scatterplot of elevational range shifts in plants in Switzerland [14] and Norway [22]. (TIFF)

S15 Fig Relationship between study duration and the proportion of species shifting inconsistently. (TIFF)

## References

[1] Walther G-R, Post E, Convey P, Menzel A, Parmesan C, Beebee TJC, et al (2002) Ecological responses to recent climate change. Nature 416: 389–395. 11919621 10.1038/416389a

[2] Parmesan C (2006) Ecological and evolutionary responses to recent climate change. Ann Rev Ecol Evol S 37: 637–669.

[3] Parmesan C, Yohe G (2003) A globally coherent fingerprint of climate change impacts across natural systems. Nature 421: 37–42. 12511946 10.1038/nature01286

[4] Chen I-C, Hill JK, Ohlemüller R, Roy DB, Thomas CD (2011) Rapid range shifts of species associated with high levels of climate warming. Science 333: 1024–1026. 10.1126/science.1206432 21852500

[5] Przeslawski R, Falkner I, Ashcroft MB, Hutchings P (2012) Using rigorous selection criteria to investigate marine range shifts. Estaur Coast Shelf S 113: 205–212.

[6] Pinsky ML, Worm B, Fogarty MJ, Sarmiento JL, Levin SA (2013) Marine taxa track local climate velocities. Science 341: 1239–1242. 10.1126/science.1239352 24031017

[7] Moran PAP (1953) The statistical analysis of the Canadian lynx cycle. Aust J Zool 1: 291–298.

[8] Post E, Forchhammer MC (2002) Synchronization of animal population dynamics by large-scale climate. Nature 420: 168–171. 12432390 10.1038/nature01064

[9] Hansen BB, Grøtan V, Aanes R, Sæther B-E, Stien A, Fuglei E, et al (2013) Climate events synchronize the dynamics of a resident vertebrate community in the high Arctic. Science 339: 313–315. 10.1126/science.1226766 23329044

[10] Pearson RG, Dawson TP (2003) Predicting the impacts of climate change on the distribution of species: are bioclimate envelope models useful? Global Ecol Biogeogr 12: 361–371.

[11] Tingley MW, Koo MS, Moritz C, Rush AF, Beissinger SR (2012) The push and pull of climate change causes heterogeneous shifts in avian elevational ranges. Glob Change Biol 18: 3279–3290.

[12] R Core Development Team (2013) R: a language and environment for statistical computing. Version 3.0.2; 2013.

[13] Archaux F (2004) Breeding upwards when climate is becoming warmer: no bird response in the French Alps. Ibis 146: 138–144.

[14] Holzinger B, Hülber K, Camenisch M, Grabherr G (2008) Changes in plant species richness over the last century in the eastern Swiss Alps: elevational gradient, bedrock effects and migration rates. Plant Ecol 195: 179–196.

[15] Lenoir J, Gégout JC, Marquet PA, de Ruffray P, Brisse H (2008) A significant upward shift in plant species optimum elevation during the 20th century. Science 320: 1768–1771. 10.1126/science.1156831 18583610

[16] Moritz C, Patton JL, Conroy CJ, Parra JL, White GC, Beissinger SR. (2008) Impact of a century of climate change on small-mammal communities in Yosemite National Park, USA. Science 322: 261–264. 10.1126/science.1163428 18845755

[17] Parolo G, Rossi G (2008) Upward migration of vascular plants following a climate warming trend in the Alps. Basic Appl Ecol 9: 100–107.

[18] Pitt NR, Poloczanska ES, Hobday AJ (2010) Climate-driven changes in Tasmanian intertidal fauna. Mar Freshwater Res 61: 963–970.

[19] Popy S, Bordignon L, Prodon R (2010) A weak upward elevational shift in the distribution of breeding birds in the Italian Alps. J Biogeogr 37: 57–67.

[20] Rowe RJ, Finarelli JA, Rickart EA (2010) Range dynamics of small mammals along an elevational gradient over an 80-year interval. Glob Change Biol 16: 2930–2943.

[21] Poloczanska ES, Smith S, Fauconnet L, Healy J, Tibbetts IR, Burrows MT, et al (2011) Little change in the distribution of rocky shore faunal communities on the Australian east coast after 50 years of rapid warming. J Exp Mar Biol Ecol 400: 145–154.

[22] Felde VA, Kapfer J, Grytnes J-A (2012) Upward shift in elevational plant species range in Sikkilsdalen, Central Norway. Ecography 35: 922–932.

[23] Bässler C, Hothorn T, Brandl R, Müller J (2013) Insects overshoot the expected upslope shift caused by climate warming. PLoS One 8: e65842 10.1371/journal.pone.0065842 23762439 PMC3676374

[24] Bodin J, Badeau V, Bruno E, Cluzeau C, Moisselin J-M, Walter G-R, et al (2013) Shifts of forest species along an elevational gradient in Southeast France: climate change or stand maturation? J Veg Sci 24: 269–283

[25] Lenoir J, Gégout J-C, Guisan A, Vittoz P, Wohlgemuth T, Zimmerman NE, et al (2010) Going against the flow: potential mechanisms for unexpected downslope range shifts in a warming climate. Ecography 33: 295–303.

[26] MacArthur RH (1972) Geographical ecology: patterns in the distribution of species Princeton: Princeton University Press.

[27] Suttle KB, Thomsen MA, Power ME (2007) Species interactions reverse grassland responses to changing climate. Science 315: 640–642. 17272720 10.1126/science.1136401

[28] Davis AJ, Jenkinson LS, Lawton JH, Shorrocks B, Wood S (1998) Making mistakes when predicting shifts in species range in response to global warming. Nature 391: 783–786. 9486646 10.1038/35842

[29] Williams JW, Jackson ST (2007) Novel climates, no-analog communities, and ecological surprises. Front Ecol Environ 5: 475–482.

[30] Gibson-Reinemer DK, Sheldon KS, Rahel FJ (2015) Climate change creates rapid species turnover in montane communities. Ecol Evol 5: 2340–2347. 26120424 10.1002/ece3.1518 PMC4475367

[31] FAUNMAP Working Group (1996) Spatial response of mammals to late Quaternary environmental fluctuations. Science 272: 1601–1606. 8662471 10.1126/science.272.5268.1601

[32] Perry AL, Low PJ, Ellis JR, Reynolds JD (2005) Climate change and distribution shifts in marine fishes. Science 308: 1912–1915. 15890845 10.1126/science.1111322

[33] Comte L, Grenouillet G (2013) Do stream fish track climate change? Assessing distribution shifts in recent decades. Ecography 36: 1236–1246.

[34] Chen I-C, Shiu H-J, Benedick S, Holloway JD, Chey VK, Barlow HS, et al (2009) Elevation increases in moth assemblages over 42 years on a tropical mountain. Proc Natl Acad Sci USA 106: 1479–1483. 10.1073/pnas.0809320106 19164573 PMC2635813

[35] Storlie C, Merino-Viteri A, Philips B, VanDerWaal J, Welbergen J, Williams S (2014) Stepping inside the niche: microclimate data are critical for accurate assessment of species’ vulnerability to climate change. Biol Lett 10: 20140576 10.1098/rsbl.2014.0576 25252835 PMC4190965

[36] Pörtner HO, Farrell AP (2008) Physiology and climate change. Science 322: 690–69. 10.1126/science.1163156 18974339

[37] Parmesan C, Ryrholm N, Stefanescu C, Hill JK, Thomas CD, Descimon H, et al (1999) Poleward shifts in geographical ranges of butterfly species associated with regional warming. Nature 399: 579–583.

[38] Foden W, Midgley GF, Hughes G, Bond WJ, Thuiller W, Hoffman MT, et al (2007) A changing climate is eroding the geographic range of the Namib Desert tree *Aloe* through population declines and dispersal lags. Diversity and Distributions 13: 245–653.

[39] IPCC (2007) Contribution of Working Group II to the Fourth Assessment Report of the Intergovernmental Panel of Climate Change, 2007 Parry ML, Canziana OF, Palutikof JP, van der Linden PJ, Hanson CE (eds). Cambridge University Press, Cambridge, United Kingdom.

[40] Araújo MB, Rozenfeld A, Rahbek C, Marquet PA (2011) Using species co-occurrence networks to assess the impacts of climate change. Ecography 34: 897–908.

[41] Boulangeat I, Gravel D, Thuiller W (2012) Accounting for dispersal and biotic interactions to disentangle the drivers of species distributions and their abundances. Ecol Lett 15: 584–593. 10.1111/j.1461-0248.2012.01772.x 22462813 PMC3999639

