# Supplementary material for: Inconsistent Range Shifts within Species Highlight Idiosyncratic Responses to Climate Warming
Source: PLoS One. 2015 Jul 10;10(7):e0132103. doi: 10.1371/journal.pone.0132103 (PMC4498742; doi:10.1371/journal.pone.0132103)
Supplement: S1 File — (DOCX) [file pone.0132103.s001.docx]

**Supporting Information File S1**
Methods for calculating the rate of range shifts

**Birds**

Archaux (2004)

Study reported a 1.3 °C increase in mean annual temperature over 25 years throughout the study region. This value was used to calculate the rate of temperature change in both study areas (1.3 °C / 25 years). Study durations at the two sites (Giffre Valley and Mont Ventoux) were 23 and 27 years, respectively, and we applied the same rate of temperature change to both areas. To calculate the range shift at each area, we used the change in mean elevation at each site between sampling periods. The change in mean elevation was divided by the time interval between sampling periods (23 or 27 years). The author reported no significant trend in precipitation during the study duration. The Giffre valley and Mont Venoux were roughly 250 km apart from one another.

Popy et al. (2010)

Study reported trends for temperature and precipitation during the breeding season, not annual means. Study reported a 1.0 °C increase in mean breeding season (March-July) temperature between 1992-1994 and 2003-2005. The duration between the midpoints of the two periods was 11 years (1.0 °C / 11 years). The study reported a decrease in mean breeding season (March-July) precipitation of 57 mm over the same interval. Study reported trends of increasing precipitation from an earlier period (1970-1995) at nearby monitoring stations.

Spatial extent of paired areas: Archaux (2004) and Popy et al. (2010)

The two study sites in southeast France (Archaux 2004) were compared to study sites in the Piedmont of northwestern Italy (Popy et al. 2010). We treated each of the three areas as separate geographic locations.

Tingley et al. 2012

Study reported trends for temperature and precipitation during the breeding season, not annual means. Species range shift data were taken from Tingley et al. (2012) and climate data for the region were taken from Chen et al. (2011). Authors use regional climate data across three sites (Millar et al. 2004^[[1]](#footnote-1)^), in which temperature increased by 0.80 °C and precipitation increased by 5.90 mm during the breeding season (May, June, and July). The interval between original surveys and resurveys varied across the three sites, so we divided the regional temperature and precipitation changes by the interval for each area, resulting in slightly different rates.

Three sites (Lassen Volcanic National Park, Yosemite National Park, and Southern Sierra) were used. Original surveys were conducted at Lassen between 1924 and 1928 (midpoint 1926), and resurveys occurred in 2007, yielding a study duration of 81 years. Original surveys in Yosemite were conducted between 1915 and 1919 (midpoint 1917), and resurveys occurred in 2004, yielding a study duration of 87 years. Original surveys at Southern Sierra occurred in 1911, and resurveys occurred in 2009, yielding a study duration of 98 years.

Spatial extent of paired areas: Tingley et al. (2012)

The three study sites are separated by more than 100 km along most of the length of the state of California, and we therefore treated them as distinct geographic areas for the purposes of comparing range shifts.

**Mammals**

Moritz et al. (2008)

Temperature changes were reported as calculated by Chen et al. (2011); the data used included minimum and maximum temperatures in January and July and a study duration of 88 years.

Precipitation data were taken from Millar et al. (2004). Precipitation changes were recorded for the period 1910-1920 (mean 417 mm/year) and 1990-2000 (mean 632 mm/year). Using the midpoints 1915 and 1995, for a duration of 80 years, the resulting rate of precipitation change was an increase of 2.7 mm/year.

Rowe et al. (2010)

Study reports changes in the summer (June-August) maximum temperature and the winter (December-February) minimum temperature at three elevational bands along the area sampled. We calculated the mean across elevation for both the maximum summer and minimum winter temperatures, then took the mean of the summer and winter temperature changes (0.685 °C). Climate data were reported from the period 1924-1929 and 2003-2008 (i.e., the three study years in each interval, 1927-1929 and 2006-2008 and the three years immediately preceding each). We took the midpoints of each six-year interval as 1927 and 2006 for a duration of 79 years. Precipitation data were reported as changes in November-April precipitation along the same elevational bands. We calculated the mean change across the three elevational bands as an increase of 18.62 mm over 79 years.

We omitted *Peromyscus truei* from our comparisons with mammals in California (Moritz et al. 2008) because its historical distribution in Nevada (Rowe et al. 2010) was not adequately characterized, making comparisons between the two eras unsuitable.

Spatial extent of paired areas: Moritz et al. (2008); Rowe et al. (2010)

Authors reported data from Yosemite National Park in the western part of central California (Moritz et al. 2008). Authors reported data from the Ruby Mountains in northeastern Nevada (Rowe et al. 2010).

**Marine invertebrates**

Pitt et al. (2010)

Study reported resurvey of transects originally surveyed in the 1950s. We used 1955 as the date for initial surveys. Resurveys were conducted in 2007-2008; we used 2008 as the date for resurveys, yielding a duration of 53 years. The authors report sea-surface temperatures have warmed at a rate of 0.0228 °C per year over the past 100 years.

Poloczanska et al. (2011)

Study reported an increase in sea temperatures of “~1.5 °C” during the past 60 years. Original surveys were conducted in the 1940s and 1950s; we used 1950 as the midpoint of this sampling. Resurveys were conducted in 2007 and 2008; we used 2008 as the midpoint. We divided the reported warming (1.5 °C) by a study duration of 58 years to calculate the rate of temperature change.

Spatial extent of paired areas: Pitt et al. (2010); Poloczanska et al. (2011)

Authors reported data collected around the coastline of Tasmania (Pitt et al. 2010). Authors reported data collected along the eastern coast of Australia (Poloczanska et al. 2011).

**Plants**

Bässler et al. (2013)

Original surveys were conducted between 1902 and 1904. Resurveys occurred between 2006 and 2007. Using the midpoints of the sampling intervals, we calculated a study duration of 104 years. Temperature and precipitation data were presented as means of 20 year periods encompassing the sampling intervals. We calculated the midpoints of these 20 year intervals as 1896 and 2001. Rates of temperature and precipitation change were taken as the total change divided by 105 years.

The authors noted the original survey of plants applied a correction factor of 70m to plant distributions located on southwest exposures, which was the exposure most common in the resurvey effort. We calculated range shifts as the total range shift listed plus 70m and used the resulting number for estimates of the rate of range shifts.

Felde et al. (2012)

Study reported mean annual temperature of -1.0 °C from 1910-1920 and -0.2 °C from 1998-2008, producing an increase of 0.8 °C over the period. The midpoint o 1910-1920 is 1915, and the midpoint for 1998-2008 is 2003, with an interval between midpoints of 88 years. We calculated the rate of temperature change as 0.8 °C / 88 years. Over the same intervals, the study reported a change in mean annual precipitation from 714 mm/year in 1910-1920 to 1,169 mm/year in 1998-2008.

The original surveys were conducted between 1922-1932, with a midpoint of 1927. Recent surveys were conducted in 2008, with a resulting interval of 81 years. We calculated changes in the upper limit of species ranges over 81 years.

Spatial extent of paired areas: Bässler et al. (2013); Felde et al. (2012)

Reported data are from Germany (Bässler et al. 2013) and Norway (Felde et al. 2012).

Holzinger et al. (2008)

We followed the methodology of Millar et al. (2004), which took the temperature change over the past 120 years of 0.6 °C. The study took place over 94 years of the 120 years in which temperatures rose by 0.6 °C; a meta-analysis (Chen et al. 2011) assumed the 94 years of the study duration equated to a temperature increase of 0.47 °C, or a rate of 0.005 °C/year. No precipitation data were reported in Holzinger et al. (2008).

Spatial extent of paired areas Bässler et al. (2013); Holzinger et al. (2008)

Reported data are from Germany (Bässler et al. 2013) and Switzerland Holzinger et al. (2008).

Spatial extent of paired areas Felde et al. (2012); Holzinger et al. (2008)

Reported data are from Norway (Felde et al. 2012) and Switzerland (Holzinger et al. 2008).

Parolo and Rossi (2008)

Study reported an increase in mean winter temperatures of 1.1 °C and an increase in summer temperatures of 1.6 °C over a 50-year period. We used the mean of the increase in summer and winter temperatures, and calculated the rate of change using over the 48 year interval between sampling year midpoints. Historic surveys occurred between 1954 and 1958 and resurveys occurred between 2003 and 2005; we used midpoints of 1956 and 2004. No precipitation data were reported.

Spatial extent of paired areas Bässler et al. (2013); Parolo and Rossi (2008)

Reported data are from Germany (Bässler et al. 2013) and Italy (Parolo and Rossi 2008).

Spatial extent of paired areas Felde et al. (2012); Parolo and Rossi (2008)

Reported data are from Norway (Felde et al. 2012) and Italy (Parolo and Rossi 2008).

Spatial extent of paired areas: Holzinger et al. (2008); Parolo and Rossi (2008)

Reported data are from Switzerland (Holzinger et al. 2008) and Italy (2008).

Bodin et al. (2013)

Study reported the rate of temperature change during 1971-2000 as 0.5 °C/decade increase. Authors reported no trend in precipitation in the study region during the study duration. Sampling occurred between 1981 and 1989 and again between 1992 and 2004. We took the mean weighted sampling year for each interval as reported by the authors (1984 and 1998),

One objective of this study was to examine the influence of stand maturation on the rate of climate-induced range shifts. We used only the data from closed forests for our analysis of range shifts because these data are least likely to be influenced by factors unrelated to climate change. If we use the data from the whole dataset (not the closed forest only) in Bodin et al. (2013), the resulting comparison with Lenoir et al. (2008) does not change appreciably. In the closed forest comparison between Bodin et al. (2013) and Lenoir et al. (2008), 32 of 68 species show inconsistent range shifts; in the whole entire comparison, 33 of 67 species show inconsistent range shifts (the numbers are not the same because some species were only recorded in closed forests).

Lenoir et al. (2008)

Temperature data were unavailable from Lenoir et al. (2008), so we used the temperature data analyzed by Chen et al. (2011), which yielded a rate of warming of 0.035 °C/year. Authors reported no trend in precipitation over the study interval.

We calculated the rate of shift in optimum elevation from the total shift reported in Lenoir et al. (2008) divided by 22 years. The initial survey period was between 1905 and 1985, with a reported mean year of survey years of 1971. The more recent survey period was between 1986 and 2003, with a mean survey year of 1993.

Spatial extent of paired areas: Bodin et al. (2013); Lenoir et al. (2008)

Authors reported data from southeastern France in Bodin et al. (2013). Authors reported data from six mountain ranges in western Europe, primarily in western France in Lenoir et al. (2008). There was some overlap in the study areas in these two studies, but Lenoir et al. (2008) included a greater geographic extent.

**Species displayed in Figure 1.**

1: *Aegithalos caudatus*; 2 *Dendrocopos major*; 3 *Dryocupus martius*; 4 *Erithacus rubecula*; 5 *Fringilla coelebs*; 6 *Garrulus glandarius*; 7 *Parus major*; 8 *Phylloscopus collybita*; 9 *Pyrrhula pyrrhula*; 10 *Regulus ignicapillus*; 11 *Regulus regulus*; 12 *Sitta europaea*; 13 *Sylvia atricapilla*; 14 *Troglodytes troglodytes*; 15 *Turdus merula*; 16 *Turdus philomelos*; 17 *Turdus viscivorus*.

1. Millar CI, Westfall RD, Delany DL, King JC, Graumlich LJ (2004) Response of subalpine conifers in the Sierra Nevada, California, U.S.A., to 20th-century warming and decadal climate variability. Arct Antarct Alp Res 36: 181-200. [↑](#footnote-ref-1)
