# Supplementary material for: Inconsistent Range Shifts within Species Highlight Idiosyncratic Responses to Climate Warming
Source: PLoS One. 2015 Jul 10;10(7):e0132103. doi: 10.1371/journal.pone.0132103 (PMC4498742; doi:10.1371/journal.pone.0132103)
Supplement: S2 File — (PDF) [file pone.0132103.s002.pdf]

Supporting Information File S2. Data and calculations for chi-square analysis.

Chi-square data for Lassen/Yosemite comparison

Data are from Tingely et al. (2012).

Table 1. Number (proportion) of bird species (*n*=45) shifting uphill or downhill in Lassen and Yosemite.

	Lassen (x-axis)	Yosemite (y-axis)
Uphill	24 (53.3%)	33 (73.3%)
Downhill	21 (46.7%)	12 (26.7%)

Table 2. Number (proportion) of bird species (*n*=45) expected and observed in four categories of paired range shifts in Lassen and Yosemite. Expected values were rounded to the nearest whole number.

	Expected (E)	Observed (O)	$(O-E)^2/E$
Uphill in Lassen, uphill in Yosemite	18 (39.1%)	18	0
Downhill in Lassen, uphill in Yosemite	15 (34.2%)	15	0
Downhill in Lassen, downhill in Yosemite	6 (12.4%)	6	0
Uphill in Lassen, downhill in Yosemite	6 (14.2%)	6	0

Code for chi-square test in R:

```
obs <- c(18,15,6,6)
chisq.test(obs, p=c(18,15,6,6)/45)

Chi-squared test for given probabilities

data:  obs
X-squared = 0, df = 3, p-value = 1
```

Chi-square data for Lassen/Southern comparison

Data are from Tingely et al. (2012).

Table 3. Number (proportion) of bird species (*n*=32) shifting uphill or downhill in Lassen and Southern.

	Lassen (x-axis)	Southern (y-axis)
Uphill	21 (65.6%)	20 (62.5%)
Downhill	11 (34.4%)	12 (37.5%)

Table 4. Number (proportion) of bird species (*n*=32) expected and observed in four categories of paired range shifts in Lassen and Yosemite. Expected values were rounded to the nearest whole number.

	Expected (E)	Observed (O)	$(O-E)^2/E$
Uphill in Lassen, uphill in Southern	13 (41.0%)	17	1.23
Downhill in Lassen, uphill in Southern	7 (21.5%)	3	2.28
Downhill in Lassen, downhill in Southern	4 (12.9%)	8	4
Uphill in Lassen, downhill in Southern	8 (24.6%)	4	2

Code for chi-square test in R:

```
obs<-c(17,3,8,4)
chisq.test(obs, p=c(13,7,4,8)/32)

        Chi-squared test for given probabilities

data:  obs
X-squared = 9.5165, df = 3, p-value = 0.02316
```

Chi-square data for Southern/Yosemite comparison

Data are from Tingely et al. (2012).

Table 5. Number (proportion) of bird species (*n*=30) shifting uphill or downhill in Southern and Yosemite.

	Southern (x-axis)	Yosemite (y-axis)
Uphill	18 (60.0%)	21 (70.0%)
Downhill	12 (40.0%)	9 (30.0%)

Table 6. Number (proportion) of bird species (*n*=30) expected and observed in four categories of paired range shifts in Southern and Yosemite. Expected values were rounded to the nearest whole number.

	Expected (E)	Observed (O)	$(O-E)^2/E$
Uphill in Southern, uphill in Yosemite	13 (42.0%)	14	0.08
Downhill in Southern, uphill in Yosemite	8 (28.0%)	7	0.13
Downhill in Southern, downhill in Yosemite	4 (12.0%)	5	0.25
Uphill in Southern, downhill in Yosemite	5 (18.0%)	4	0.20

Code for chi-square test in R:

```
obs<-c(14,7,5,4)
chisq.test(obs, p=c(13,8,4,5)/30)

Chi-squared test for given probabilities

data:  obs
X-squared = 0.6519, df = 3, p-value = 0.8844
```

Chi-square data for southeastern France/western France comparison

Data in southeastern France are from Bodin et al. (2013). Data in western France are from Lenoir et al. (2008).

Table 7. Number (proportion) of bird species (*n*=67) shifting uphill or downhill in southeastern France and western France.

	Southeastern France (x-axis)	Western France (y-axis)
Uphill	39 (58.2%)	46 (68.7%)
Downhill	28 (41.8%)	21 (31.3%)

Table 8. Number (proportion) of bird species (*n*=30) expected and observed in four categories of paired range shifts in Southern and Yosemite. Expected values were rounded to the nearest whole number.

	Expected (E)	Observed (O)	$(O-E)^2/E$
Uphill in SE France, uphill in W France	27 (40.0%)	27	0
Downhill in SE France, uphill in W France	19 (28.7%)	19	0
Downhill in SE France, downhill in W France	9 (13.1%)	9	0
Uphill in SE France, downhill in W France	12 (18.2%)	12	0

Code for chi-square test in R:

```
obs<-c(27,19,9,12)
chisq.test(obs, p=c(27,19,9,12)/67)

        Chi-squared test for given probabilities

data:  obs
X-squared = 0, df = 3, p-value = 1
```
