# Supplementary figures and images for: Inconsistent Range Shifts within Species Highlight Idiosyncratic Responses to Climate Warming
Source: PLoS One. 2015 Jul 10;10(7):e0132103. doi: 10.1371/journal.pone.0132103 (PMC4498742; doi:10.1371/journal.pone.0132103)

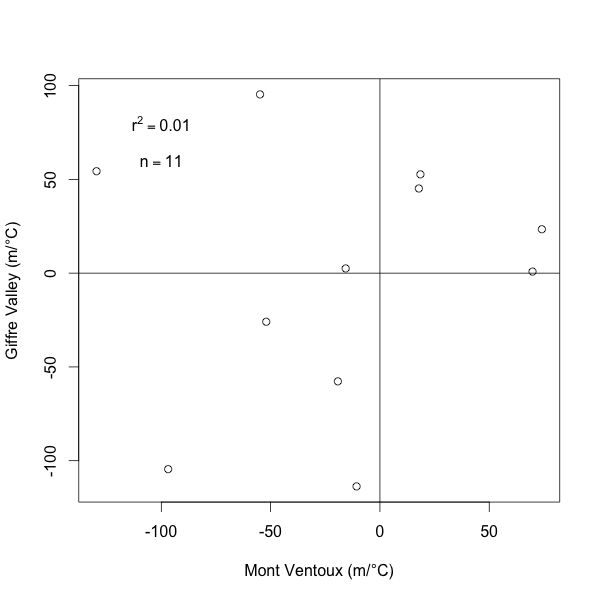

Supplement: S1 Fig — (TIFF) [file pone.0132103.s003.tiff]

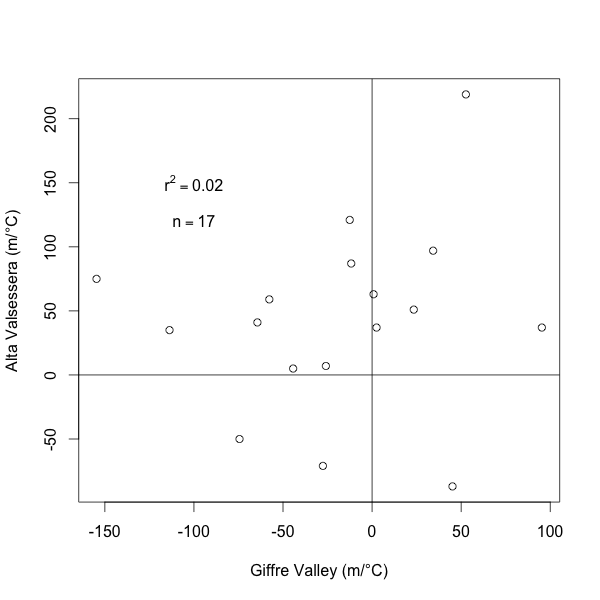

Supplement: S2 Fig — (TIFF) [file pone.0132103.s004.tiff]

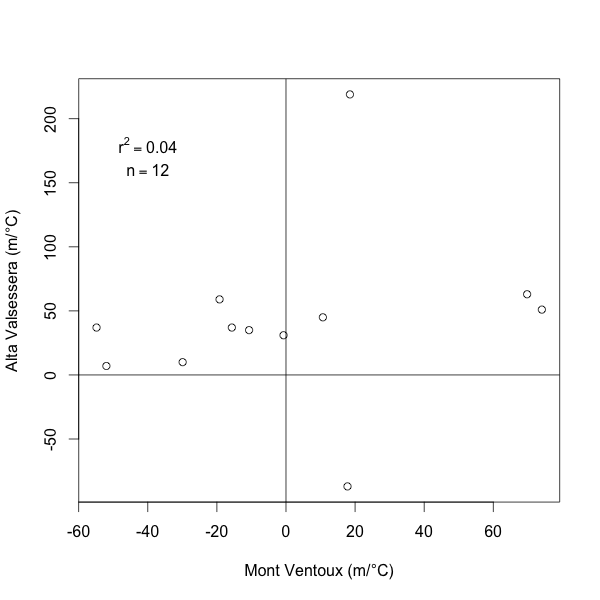

Supplement: S3 Fig — (TIFF) [file pone.0132103.s005.tiff]

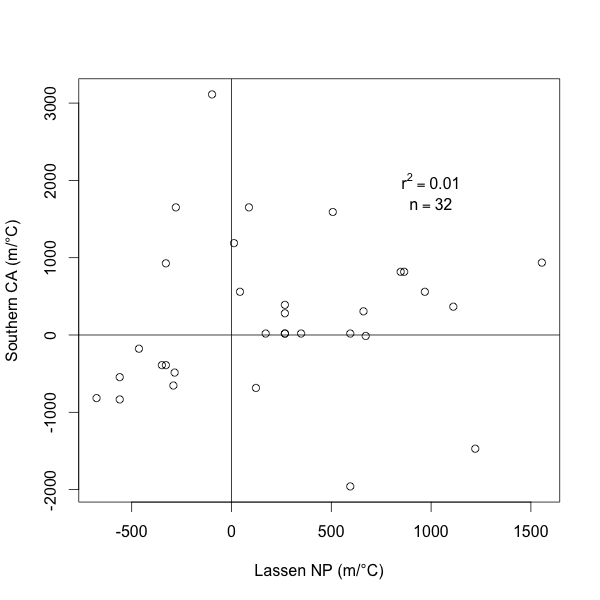

Supplement: S4 Fig — (TIFF) [file pone.0132103.s006.tiff]

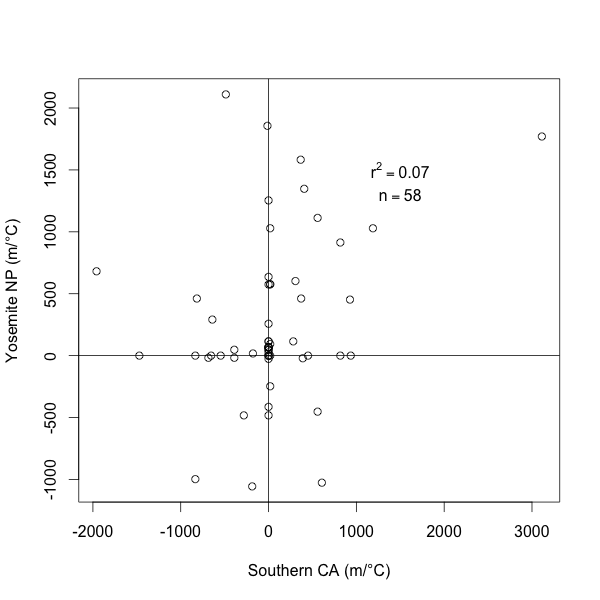

Supplement: S5 Fig — (TIFF) [file pone.0132103.s007.tiff]

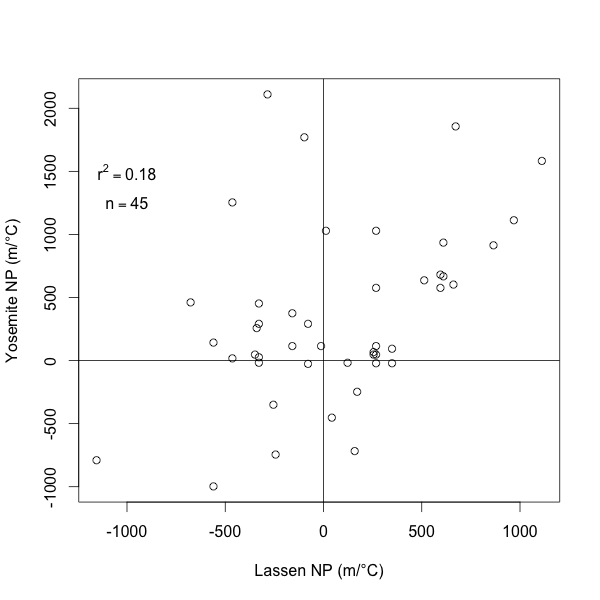

Supplement: S6 Fig — (TIFF) [file pone.0132103.s008.tiff]

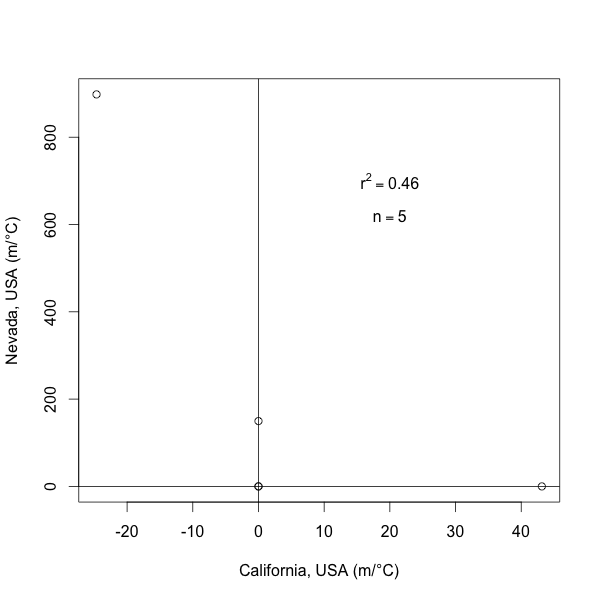

Supplement: S7 Fig — (TIFF) [file pone.0132103.s009.tiff]

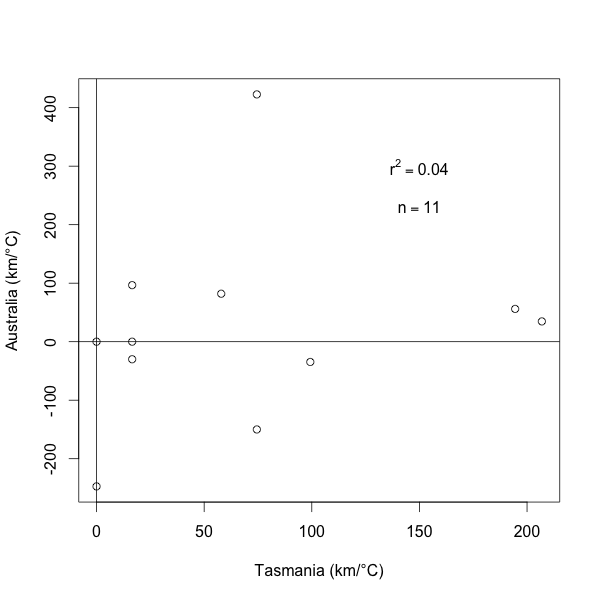

Supplement: S8 Fig — (TIFF) [file pone.0132103.s010.tiff]

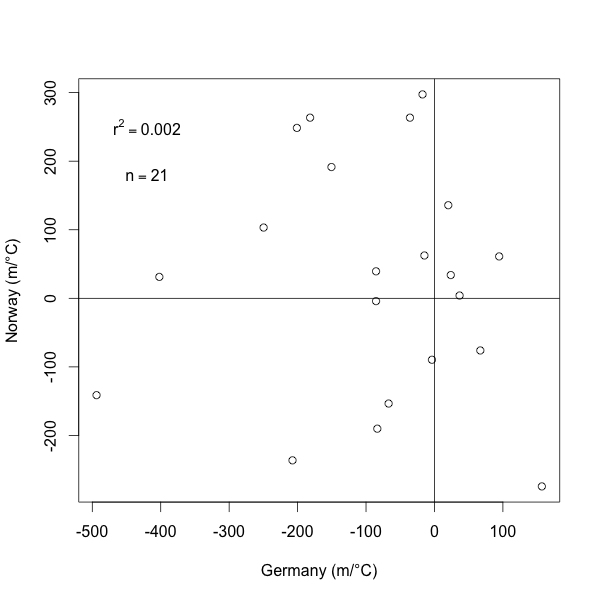

Supplement: S9 Fig — (TIFF) [file pone.0132103.s011.tiff]

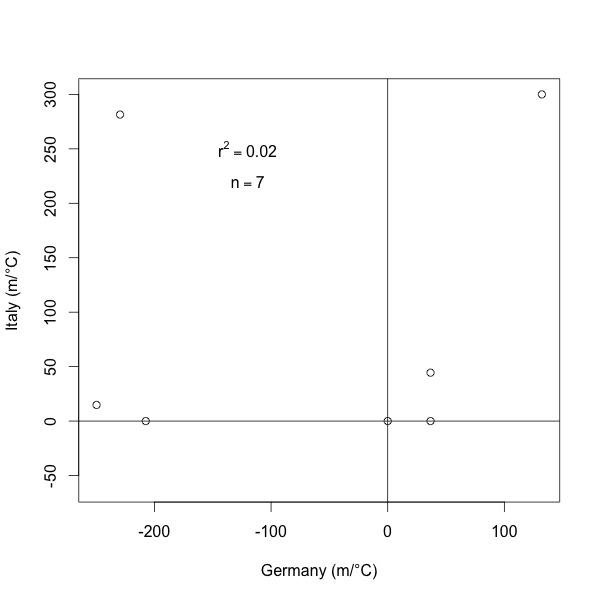

Supplement: S10 Fig — (TIFF) [file pone.0132103.s012.tiff]

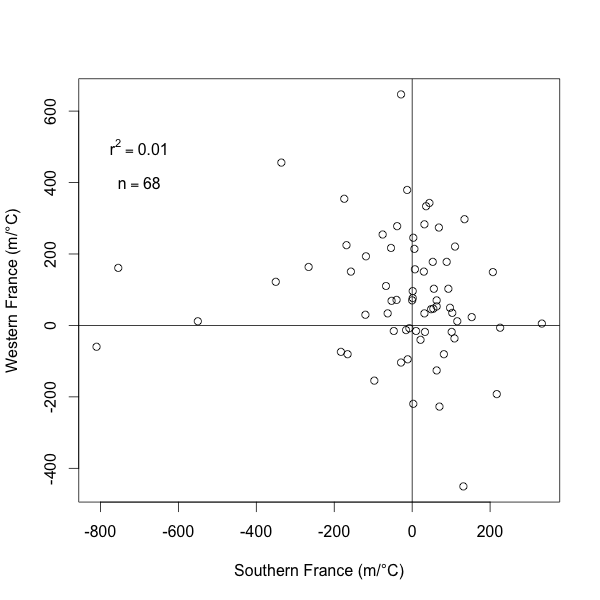

Supplement: S11 Fig — (TIFF) [file pone.0132103.s013.tiff]

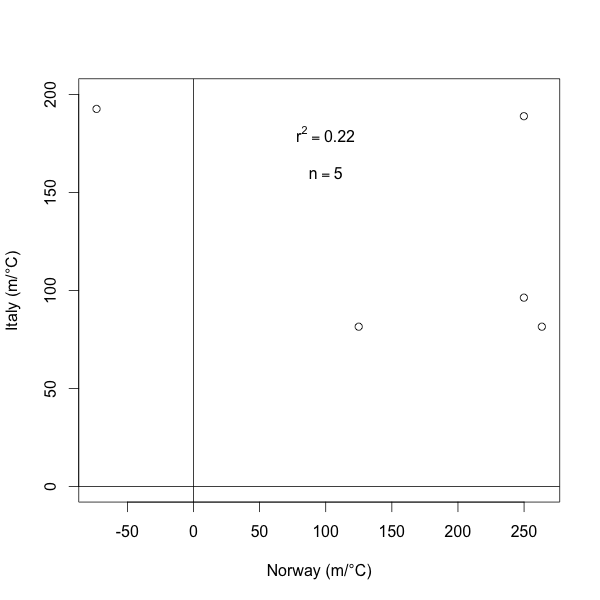

Supplement: S12 Fig — (TIFF) [file pone.0132103.s014.tiff]

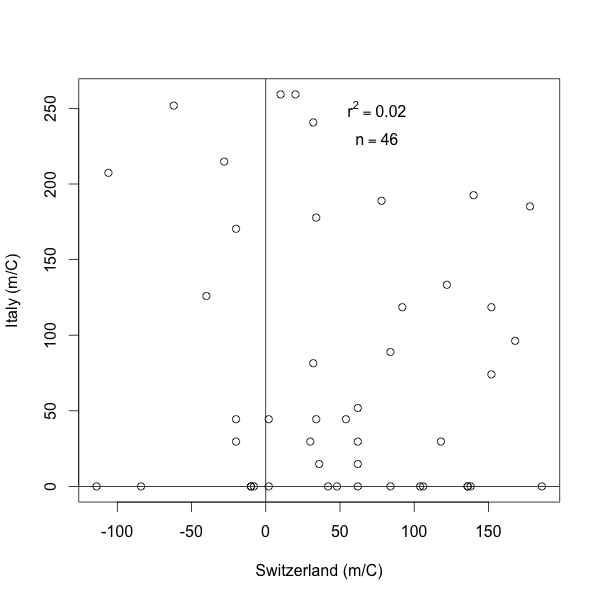

Supplement: S13 Fig — (TIFF) [file pone.0132103.s015.tiff]

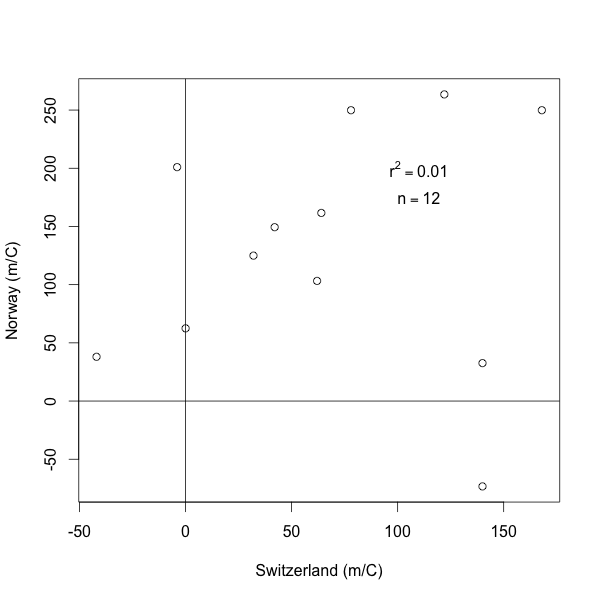

Supplement: S14 Fig — (TIFF) [file pone.0132103.s016.tiff]

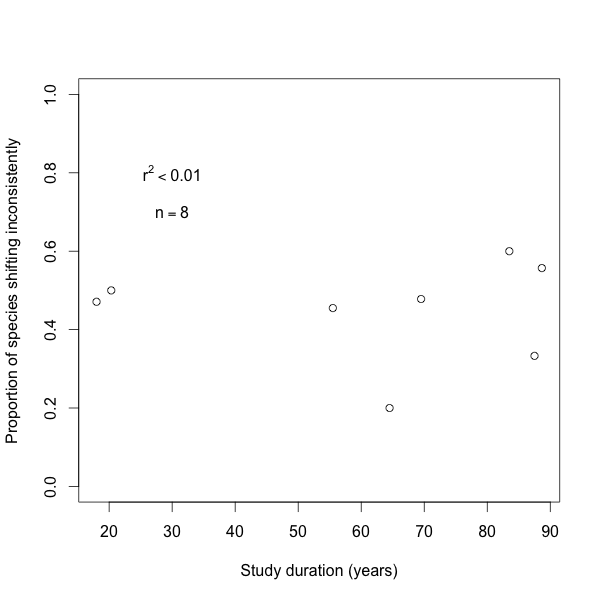

Supplement: S15 Fig — (TIFF) [file pone.0132103.s017.tiff]
